# Generation Scotland: the Scottish Family Health Study; a new resource for researching genes and heritability

**DOI:** 10.1186/1471-2350-7-74

**Published:** 2006-10-02

**Authors:** Blair H Smith, Harry Campbell, Douglas Blackwood, John Connell, Mike Connor, Ian J Deary, Anna F Dominiczak, Bridie Fitzpatrick, Ian Ford, Cathy Jackson, Gillian Haddow, Shona Kerr, Robert Lindsay, Mark McGilchrist, Robin Morton, Graeme Murray, Colin NA Palmer, Jill P Pell, Stuart H Ralston, David St Clair, Frank Sullivan, Graham Watt, Roland Wolf, Alan Wright, David Porteous, Andrew D Morris

**Affiliations:** 1 University of Aberdeen, Department of General Practice and Primary Care, Foresterhill Health Centre, Westburn Road, Aberdeen, UK; 2 University of Aberdeen, Department of Pathology, University Medical Buildings, Foresterhill, Aberdeen, UK; 3 University of Aberdeen, College of Life Sciences and Medicine, Polworth Building, Aberdeen, UK; 4 University of Dundee, Biomedical Research Centre, Level 5, Ninwells Hospital, Dundee, UK; 5 University of Dundee, Community Health Sciences Division, MacKenzie Building, Kirsty Semple Way, Dundee, UK; 6 University of Dundee, Division of Medicine and Therapeutics, Level 7, Ninewells Hospital and Medical School, Dundee, UK; 7 University of Dundee, Tayside Centre for General Practice, Kirsty Semple Way, Dundee, UK; 8 University of Dundee, Health Informatics Centre, MacKenzie Building, Kirsty Semple Way, Dundee, UK; 9 University of Edinburgh, Western General Hospital, Rheumatic Diseases Unit, Edinburgh, UK; 10 University of Edinburgh, Old Surgeons Halls, High School Yards, Edinburgh, UK; 11 University of Edinburgh, Department of Psychiatry, The Royal Edinburgh Hospital, Morningside Park, Edinburgh, UK; 12 University of Edinburgh, Division of Community Health Sciences, Medical School, Teviot Place, Edinburgh, UK; 13 University of Edinburgh, Department of Psychology, 7 George Square, Edinburgh, UK; 14 University of Edinburgh, Molecular Medicine Centre, Western General Hospital, Crewe Road, Edinburgh, UK; 15 University of Edinburgh, MRC Human Genetics Unit, Western General Hospital, Crewe Road, Edinburgh, UK; 16 University of Glasgow, BHF Glasgow Cardiovascular Research Centre, 126 University Place, Glasgow, UK; 17 University of Glasgow, Division of Cardiovascular & Medical Sciences, Glasgow, UK; 18 University of Glasgow, Robertson Centre for Biostatistics, 15 University Gardens, Glasgow, UK; 19 University of Glasgow, Division of Community-Based Sciences, Glasgow, UK; 20 University of Glasgow, Institute of Medical Genetics, Yorkhill, Glasgow, UK

## Abstract

**Background:**

Generation Scotland: the Scottish Family Health Study aims to identify genetic variants accounting for variation in levels of quantitative traits underlying the major common complex diseases (such as cardiovascular disease, cognitive decline, mental illness) in Scotland.

**Methods/Design:**

Generation Scotland will recruit a family-based cohort of up to 50,000 individuals (comprising siblings and parent-offspring groups) across Scotland. It will be a six-year programme, beginning in Glasgow and Tayside in the first two years (Phase 1) before extending to other parts of Scotland in the remaining four years (Phase 2). In Phase 1, individuals aged between 35 and 55 years, living in the East and West of Scotland will be invited to participate, along with at least one (and preferably more) siblings and any other first degree relatives aged 18 or over. The total initial sample size will be 15,000 and it is planned that this will increase to 50,000 in Phase 2. All participants will be asked to contribute blood samples from which DNA will be extracted and stored for future investigation. The information from the DNA, along with answers to a life-style and medical history questionnaire, clinical and biochemical measurements taken at the time of donation, and subsequent health developments over the life course (traced through electronic health records) will be stored and used for research purposes. In addition, a detailed public consultation process will begin that will allow respondents' views to shape and develop the study. This is an important aspect to the research, and forms the continuation of a long-term parallel engagement process.

**Discussion:**

As well as gene identification, the family-based study design will allow measurement of the heritability and familial aggregation of relevant quantitative traits, and the study of how genetic effects may vary by parent-of-origin. Long-term potential outcomes of this research include the targeting of disease prevention and treatment, and the development of screening tools based on the new genetic information. This study approach is complementary to other population-based genetic epidemiology studies, such as UK Biobank, which are established primarily to characterise genes and genetic risk in the population.

## Background

Most medical disorders of public health importance have a significant heritable component. This includes cancer, heart disease/stroke and mental health (the three current health priorities in Scotland) [1], as well as a wide range of chronic or currently untreatable causes of ill health. With the current changes in the demographic profile of Western society, including Scotland [2], there will be a steady increase in the prevalence of chronic disease over the next two decades. With few exceptions, however the specific nature of the heritable risk factors for chronic disease remains elusive. As a result of the successful completion of the human genome project [3-5], there is an opportunity to identify and characterise these heritable (genetic) risk factors. In the long term this will inform and potentially improve disease surveillance, treatment optimisation, avoidance of adverse drug events and prediction of response to therapy (pharmacogenetics), health care planning and drug discovery (pharmacogenomics) [6-8]. The benefits of such research to individuals and society are potentially substantial.

### Funding and collaborations

Generation Scotland (GS) is a collaborative initiative between the Universities of Aberdeen, Dundee, Edinburgh, Glasgow and St Andrews, and the Medical Research Council Human Genetics Unit, the National eScience Centre, the Scottish School of Primary Care, and the National Health Service (NHS) Scotland. It is funded by the Scottish Funding Council (formerly Scottish Higher Education Funding Council), and aims to promote research into genetics and healthcare [9]. Generation Scotland: the Scottish Family Health Study (GS:SFHS) is the first major study undertaken by GS, and has been funded by the Scottish Executive Health Department, Chief Scientist Office (SEHD CSO) under their Genetics and Healthcare Initiative [10], to bring together existing and complementary strengths in Scotland in the arena of genetics as applied to healthcare.

## Aims and objectives

The aim of GS:SFHS is to establish a new, large, family-based intensively-phenotyped cohort recruited from the general population across Scotland, as a resource for studying the genetics of health areas of current and projected public health importance. DNA and non-identifiable information from this cohort will be made available to researchers in Scotland and international collaborators. It will be a six-year fieldwork programme, beginning in Glasgow and Tayside in the first two years (Phase 1) before extending to other parts of Scotland in the remaining four years (Phase 2).

The objectives are:

1. **Recruitment**. To recruit and phenotype a family-based cohort from across Scotland, allowing identification of genetic variants relevant to the pathogenesis of common complex diseases (including cardiovascular disease, age-related cognitive decline, and mental illness) and pharmacogenetics research.

2. **Public Understanding**. To conduct an early and sustained public consultation programme to understand and explain the public reaction to genetics in healthcare, and their reaction to participation in research.

3. **Research capacity**. To create a multi-institutional and NHS collaboration across Scotland that will share knowledge and best practice in human genetics research.

4. **Health informatics**. Through this collaboration, to create a nationwide research platform in emerging technologies of health informatics in genetics research, including the linkage of study data to "real-time" routine healthcare data on an individual basis.

5. **NHS integration**. To work with NHS Clinical Genetics colleagues to develop computer-delivered education on genetics for healthcare professionals in Scotland.

6. **Exemplar studies**. To conduct specific research projects with identified protocols in the areas of cardiovascular disease, mental health, osteoporosis and pharmacogenetics, using the resource that will be established.

This paper describes the methodology for the first of these objectives, currently in its pilot phase, though it is important that all of the objectives are closely integrated. In particular, findings from the public consultation will contribute directly to continuous development of the materials and methods used to recruit the cohort, and the study will undergo periodic review in light of the findings of this parallel component. This will include study of potential participants' views on recruitment, consent, withdrawal, feedback, confidentiality, storage of and access to bioinformation, commercialisation, governance and other associated ethical, legal and social issues. This article therefore discusses the structure and form of a nascent GS.

## Methods and design

### Identification and recruitment of participants

This is a family-based study, recruiting participants and their relatives mainly through primary care. Recruitment of general practices is facilitated by Scottish Practices and Professionals Involved in Research (SPPIRe) [11]. Potential participants are identified from the registers of collaborating general practices via the Community Health Number, a unique identifying number allocated to every individual in Scotland who is registered with a general practitioner (GP), ie approximately 96% of the population. They are eligible to participate if they are aged between 35 and 55 years and have at least one first degree relative aged 18 years or over, and at least one full sibling group (the larger the better) in the participating family group.

An independent party, based in the NHS, generates a list of eligible people registered with each collaborating general practice, from the Scottish NHS register (known as the Community Health Index (CHI)). The names of all potential participants are screened by their GP, and individuals whom it might be inappropriate to approach (such as those with a serious or terminal illness, or those unable to consent) are excluded. Letters of invitation to eligible participants are generated on practice headed note-paper and signed by one of its GP Principals. These letters are dispatched by the independent party by post, with up to two reminders as required. This invitation is for agreement to discuss the study with family members with a view to possible participation. When the eligible person returns the tear-off slip agreeing to be contacted by the research team the individual's details are sent from the independent party to the study team.

In addition, targeted approaches in the Tayside area will be made to potentially eligible individuals whose details are held on the Walker Birth Cohort database [12]. This is a database of over 48,000 births in Dundee between 1952 and 1966, identified by CHI numbers, and therefore with current information about family structure and location. This provides the opportunity to approach individuals and their families simultaneously, with the ability to maximise the efficiency of recruitment by targeting larger local families in the first instance.

Upon receiving their permission, potential participants are contacted by a member of the research team to ensure that participants understand the study, that all demographics and details are accurate, and to discuss participation in the study with the relevant first degree relatives (including at least one sibling group). The names and contact details are requested of all first degree relatives who have verbally indicated, to the individual initially contacted, their willingness to be approached by the research team. These relatives are then also contacted by telephone by the study team. Each relative contacted is invited to discuss with and identify further first degree relatives, and so on, with the aim of creating a "snowball" sampling effect.

Similar methods of approaching and recruiting participants and their relatives have been successfully used in other studies in which several of the co-applicants have been closely involved. These include the British Genetics of Hypertension (BRIGHT) study [13] and "Family and population genetic studies in major mental illness" (D Blackwood *et al*). Although it is the main method of approach and recruitment, it will be augmented by a programme of communication and publicity about the study. Throughout this programme, individual families will be invited to volunteer directly for the study, by contacting the research team. They will be able to participate if the family includes at least one sibling pair.

### Data to be collected

Following verbal consent, individuals and their relatives who agree to consider participation in the clinical study are sent a copy of the Participant Information Leaflet (PIL), a consent form, and a pre-clinic questionnaire (PCQ), and arrange for attendance at a research clinic at a mutually convenient time and location. They are asked to bring these and any medications taken regularly, to the clinic visit. If possible, this appointment is at a time that allows collection of a fasting blood sample. The feasibility of this is being reviewed as the study progresses.

On attendance at the clinic the consent form is discussed and completed prior to any sample or medical details being taken. The participant then hands over the completed PCQ, with the opportunity for clarification of any items with the research nurse, has clinical and interview measurements made, and provides blood and urine samples, all according to detailed Standard Operating Procedures (SOPs). This visit lasts between 90 minutes and two hours.

The data collected aim to maximise the research benefit of each individual's attendance while retaining efficiency and acceptability. As well as the PCQ, they include biometric measurements, tests of cognitive function, questionnaires relating to personality traits and psychological distress, and screening for major mental illness (Table 1). Validated questionnaire instruments and clinical SOPs are used where these are available. The clinic processes are subject to regular internal monitoring for adherence to SOPs and protocol.

**Table 1:** Data collected from participants in GS:SFHS

1. **Pre-clinic questionnaire**
a. Demographic details
b. Occupational history
c. Lifestyle – smoking, alcohol, diet, exercise and participation in sports
d. Personal and family medical history
e. Rose angina questionnaire
f. Musculoskeletal questions – including chronic pain, fractures, joint replacements, HRT use, age at
menarche and menopause
g. Current drug history, including prescribed and some non-prescribed,

2. **Physical measurements**
a. Height, weight
b. Blood Pressure × 2, resting pulse
c. Ankle brachial pressure index
d. Spirometry
e. Electrocardiograph
f. Bioimpedance

3. **Personality, Cognitive function, and Psychological distress measurements**
a. Eysenck Personality Questionnaire Revised Short Form[27]
b. Logical Memory from the Wechsler Memory Scale III[28]
c. Digit Symbol from the Wechsler Adult Intelligence Scale III[29]
d. Verbal Fluency[30]
e. Mill Hill Vocabulary Scale[31]
f. General Health Questionnaire-28[32]

4. **Mental health measurements**
a. SCID Brief screening interview for major depression[33]

5. **Biological samples**
a. Blood: EDTA for DNA – 2 × 4.7 ml, 1 × 9 ml;
Clot activator with gel separator for serum – 3 × 5 ml
Fluoride oxalate for glucose – 1 × 2 ml
b. Urine: Stored for future proteomics analysis

Demographic, clinical and biological data are entered initially on a paper clinical reporting form (CRF), but kept separately from any personal details that would allow identification of individuals, using a barcode system. The PCQ and CRF are scanned into an electronic database, with detailed in-built validity checks of data recording. Feedback is given to participants and, with their permission, their GP, in the form of basic clinical information (height, weight, body mass index, blood pressure, serum cholesterol and glucose, and smoking status). ECGs are reported centrally, and summarised results are also fed back to GPs, with permission. Detailed results of these are returned to the research team for entry to the database, and summarised results are fed back to GPs (with the participants' consent), along with basic clinical recommendations based on these.

### Planned analysis, statistical power

The primary objective of this study is to establish the cohort and database as a resource for future analysis. It is important to maximise the utility of the resource, and some general principles of analysis have been determined. We propose to adopt both variance components linkage and regression approaches to the genetic linkage analysis of continuous quantitative trait data (QT) since these have been shown to be powerful approaches [14]. This will be complemented by using parent-offspring groups to carry out family-based association analysis in the regions of the QT loci (QTLs) identified in the linkage study. The power of variance components linkage analysis varies, according to the specific genetic and environmental effects and components of variance used to model the phenotypic mean, and the resemblance between pedigree members. A precise estimate for the statistical power of the study requires precise details of the family structure of the sample, which is not yet known. Nevertheless, a general statement regarding the power of the study can be made. Calculations and simulations performed by Williams and Blangero [14] have shown that each of the following family structures would have 80% asymptotic power to detect a QTL of heritability 0.10 (0.15) [0.2] with a LOD score of greater than 3, indicating evidence of linkage:

15,000 (7,500) [4,000] sibpairs, thus requiring recruitment of 30,000 (15,000) [8,000] individuals

4,700 (2,400) [1,333] sibtrios thus requiring recruitment of 14,400 (7,200) [4,000] individuals

2,400 (1,200) [625] sibquads thus requiring recruitment of 9,600 (4,800) [2,500] individuals

78 (38) [21] pedigrees each of 48 members thus requiring recruitment of 3,750 (1,800) [1,000] individuals

Our recruitment is based on families with a minimum of a sibpair. However, we seek to recruit all siblings within families that are recruited, and aim to recruit as many sibtrios as possible. Recruitment of parents and adult offspring will further increase study power and will permit future family-based association studies and haplotype analysis. We therefore also seek to recruit one or both parents, wherever possible. If we take the power estimates for sibtrios as our main estimate then in ideal circumstances we will require to recruit 14,400 (7,200) sibs to be able to identify a QTL with an effect corresponding to QTL heritability of 10% (15%). The proposed cohort size over the first 2 years will effectively be between these two estimates. Actual study power will be increased by the inclusion of parental data and will increase in proportion to our success in recruiting some larger pedigrees but will be decreased by any genotyping errors (errors in family relationship data should be identified as part of the genetic analysis), QT measurement error and imperfect reliability (repeatability) of QT measures. We believe that a study power calculation based on that for sibtrios is a reasonable compromise and suggest that the study will have 80% power to detect a QTL heritability of between 10% and 15%. We will further maximise study power by selecting traits for study based on their known (high) heritability and repeatability. If we are successful in targeting and recruiting larger families then the above power calculation will be very conservative and significantly underestimate the true study power. As recruitment rises towards 50,000 in years three to six this will give study power to detect QTL heritability of between 5% and 10%.

### Data and sample storage

Questionnaire and interview data are optically scanned in to a secure database with a unique study number identifying each individual. Biochemical samples are processed by local NHS laboratories, and the data entered on to the same secure database. Further biochemical samples (serum and urine) are processed locally in research labs and archived for future analysis. A 9 ml EDTA blood sample will be delivered (in batches of frozen tubes) to the Wellcome Trust Clinical Research Facility in Edinburgh [15], for DNA extraction to master and working stocks and further dissemination to researchers around Scotland and internationally, for specific hypothesis-driven analyses and with proper ethical and other relevant approvals. Precise access agreement is being defined according to Wellcome Trust and Medical Research Council guidance [16].

### IT infra-structure and data linkage

An important part of GS:SFHS is the development of an IT infra-structure that can support the research, from identification of potential participants to linkage of data with other useful databases. This both facilitates the study and maximises its value. A suite of Windows-based applications and services, implemented with web services, provides access to IT facilities for identification, approach and recruitment of subjects, appointments, consent, storage of paper-based data, and feedback of clinical information. Participants are identified by a unique barcode, with systems to ensure efficient and confidential handling of all data.

Through the CHI numbers of participants, and by a process of encryption, study data will be linked to a number of routine datasets, including virtually all formal healthcare activity (Table 2). Consent for this is sought at the time of recruitment. This allows the baseline phenotype data to be extended from that which will be collected specifically for the study, and will also allow longitudinal collection of real-time health data for the foreseeable future. This linkage and data collection is already possible in the Tayside area, and extension of this facility to the rest of Scotland will be part of the developmental process of this study. One key methodological resource relevant to the study is the ability to link to all prescribed drugs dispensed to patients in Tayside, and increasingly throughout Scotland. The confirmed ability [17,18] to link large and comprehensive clinical datasets to extensive drug prescription records at an individual level, over a ten year period, is not available anywhere else in the world.

**Table 2:** Healthcare datasets with which GS:SFHS data will be linked.

**DATASET**	**DESCRIPTION**	**COVERAGE**	**DATES**
**CHI dataset**	NHS number (unique personal identifier) (Includes DOB/death)	Scotland wide, currently 5.6M people	1980-present
**ISD SMR1 record**	All hospital admissions – ICD9/10 coded	Scotland wide, currently 750k p.a.	1980-present
**MEMO- Rx**	Regional prescribing of all pharmacies	Tayside wide	1993-present
**Mobile Eye Screening**	Mobile digital retinal photographic unit	All GPs; total 33,000.	1991-present
**Tayside Biochemistry**	All biochemical investigations	Tayside wide, 25M data items	1993-present
**Retinal Laser Clinic**	Regional laser clinic	Tayside	1998-present
**DARTS Validation**	All diabetes patients	Tayside wide-all 78 GP Practices	1994-present
**Cardiac outcomes**	Coronary Care Unit admissions, echocardiography, Exercise Tolerant Tests, etc.	Tayside	1997-present
**Walker Database**	Tayside birth records from 1952–66	Tayside, 50k births; 73 data items	1952–1966
**ISD SMR 2, 6, 10,11**	Maternity, cancer, neonatal, child health records	Tayside	1980-present
**GPASS data**	Primary care clinical and prescribing data	Being rolled out to 65 GP practices	ongoing
**HEARTS**	All cardiac admissions	Tayside wide	1996-present
**Endoscopy**	All clinic data	Tayside	1990-present
**Stroke**	All Tayside strokes	Tayside	1993-present
**Asthma**	A cohort of 4,500 asthma patients	Tayside	1990-present

**Note**. Although some of these are currently only available in the Tayside region, it is intended that the capacity will be exported across the rest of Scotland as part of this study.

## Ethical issues

All components of GS:SFHS, including the protocol and written study materials, have received formal, national ethical approval from NHS Tayside Research Ethics Committee (REC reference number 05/S1401/89). In addition local approval has been obtained from NHS Glasgow Research Ethics Committee, and from NHS Glasgow and NHS Tayside Research and Development Offices, as is required.

The study aims to minimise several risks to participants.

### Initial contact and recruitment

1. Direct researcher contact maximises subject accrual but at the risk of compromising privacy and confidentiality. The use of an independent party to work between the research team and participating general practices is designed to minimise the risk to the privacy of potential participants. We need to ascertain what the actual recruitment rate will be, and the incorporation of the public consultation study will ascertain what issues are likely to affect participation, and how these might be addressed.

2. Heightened risk of disclosing confidential information. No information is given by the research team to probands or relatives about each other, and only basic lifestyle and health information will be fed back to participants. Separate discussions between the research nurse and both the proband and the relative(s) maintain confidentiality, and allow individual concerns to be addressed and a private and frank discussion about participation to ensue.

3. Because it is impossible to know what precise purposes the resources will be used for in the future, fully informed consent for the use of data and DNA cannot be obtained. Because of the need to maintain participant anonymity and confidentiality, the logistical difficulties of re-tracing participants and the annoyance factor of persistent re-consenting, currently "blanket" consent is sought, for full research and commercialisation purposes.

### Participation

1. Despite this being a family study, individual confidentiality remains paramount and no medical information is given to individuals or relatives about themselves or each other, apart from personal lifestyle and basic clinical information to individuals who request this at the time of collection.

2. We emphasise that participation is at all times voluntary. Withdrawal will be allowed at any time, upon which all data and samples, that have not been analysed, relating to the withdrawing participant will be destroyed.

3. Bias will be introduced into both the clinical and consultation study in recruitment of family members by the proband. Family is not purely about biological connections but social and kinship networks. The design offers no option other than to go with the family's choice in who can participate, and their stated biological relationships with each other.

4. No genetic feedback will be given to participants; this is a project that aims to construct a database for research purposes and does not purport to offer genetic tests either to individuals or populations.

5. Access to the samples and data by third parties will be reviewed by GS:SFHS senior management groups, subject to an independent governance system whose details are to be finalised in light of the public consultation study and external stakeholder advice, and NHS Research Ethics Committees. Data will only be made available to outside parties after personal identifying information has been removed and/or securely encrypted.

The REC will be informed of progress and proposed modifications to the protocol. In addition, an independent GS Advisory Board has been established under the auspices of SEHD CSO, and will monitor the study's procedures, protocols and data storage and access. This will be analogous to a Data Monitoring Committee in a clinical trial, and will have right of access to all study materials (anonymised when appropriate).

## Discussion

Unlike general population biobank studies, such as UK Biobank [19], GS:SFHS does not focus on gene:environment interactions, and is not powered for this. Its main focus – on identifying gene loci associated with important QTs – means that it will complement UK Biobank and other studies, and much of the data collection is designed with this in mind.

### Scottish advantages

The Scottish population (currently 5.1 million approximately [20]) is ideally suited to genetic epidemiological studies of many important diseases, including cardiovascular, cerebrovascular, metabolic diseases, mental illness and cancer. The overall incidence, prevalence and mortality from these diseases is high and premature deaths are relatively common [21]. In addition, Scotland has a high prevalence of adverse lifestyle risk factors [22], which facilitate studies of the interactions between lifestyle and genetic factors. A further advantage of Scotland is its relatively stable population. A complete follow-up is most likely to be achieved if the study recruits people who continue to reside in the same geographical area and remain registered with the same general practitioner. The annual migration rate from Scotland is less than 1%, and there is clear evidence that emigration from Scotland to other areas of the United Kingdom is lower than any other region [23]. In addition past experience has shown that the Scottish people are supportive of community based cohort studies [13].

### Unique features of GS:SFHS

GS:SFHS is one of a growing number of large DNA collections or biobanks across the world. Two important unique characteristics of GS:SFHS will be (i) the large general population family-based nature, and (ii) the ability to link individual data with detailed past and future health records. It is also unique for its inclusion of detailed cognitive function phenotype information, and its collection of data on serious mental illness, both of which will provide rich opportunities to study the genetics of these health areas.

### Data linkage

The key parts of GS:SFHS are the desire to intensively phenotype family members at baseline and to follow up participants through routine data systems and to seek their consent for further fieldwork. Scotland is uniquely well-placed to allow linkage of participants' collected data with routine data from the NHS because of the widespread use of unique CHI numbers linking individuals, through the CHI, with many other NHS datasets. A bioinformatic infrastructure is well-developed to support research that maximizes the value of this while retaining the anonymity of participants. Tayside is currently the only region in the UK where the CHI number is used as the patient identifier in the virtually all health care activities, from primary to tertiary care, from birth to death, and is added routinely to all research datasets in order to enable further phenotype enrichment. A core aim of the SFHS is to export expertise in Tayside to other centres in Scotland.

### Advantages of a family based approach

Linkage analysis has proven to be very effective for identifying genes concerned with rare Mendelian disorders and Mendelian forms of genetically complex ones. Thus, it is the major strategy used for identification of genetic variants of large effect.

However large genetic effects tend to result in selection against these alleles, therefore causing only rare diseases or rare forms of common diseases. The low power to detect genetic variants of small effect has suggested that this approach may be much less effective for common complex diseases. However, recent pedigree-based studies carried out by deCODE Genetics in Iceland have reported promising results with denser marker scans and larger pedigrees than in past studies [24]. GS:SFHS therefore aims to recruit large numbers of families using a family-based approach.

The focus of the study is to identify genetic variants of moderate rather than large (likely to be already discovered) or small (unlikely to be detectable by this approach) effect sizes. It is likely that genetic factors with large and moderately large effects on complex traits or disease risk code for proteins involved in important physiological mechanisms. In addition therefore, they are likely to be important both in terms of our understanding of pathological mechanisms and of their potential to lead to new prevention or treatment strategies.

This pedigree or family-based approach has a number of other advantages:

• the ability to make use of familial correlations to increase power using strategies such as the EDAC ("extreme discordant and concordant") design.

• the ability to obtain information on genetic haplotypes (most readily determined with family data, particularly over extended genetic distances, despite recent improvements in prediction).

• the ability to carry out a family based association approach including using the transmission-disequilibrium test (TDT) by comparing transmitted with non-transmitted parental alleles.

• the ability to provide parent-of-origin information (for example, the relationship between early life events and adult diseases may involve maternally or paternally imprinted loci, such as IGF2).

### Existing family-based DNA collections

Most family-based collections have been affected sibpair studies [24], for a number of specific diseases. Most of these studies have been individually small but have gained power through international collaborations. There have been far fewer sibpair studies which have focused on measurement of a wide range of disease-related QTs, as this study will, and none of the proposed scale of GS:SFHS. The other major family-based design has been twin studies based around a number of national or regional twin registries in northern European countries, and these are being developed further through Genome EU Twins [25]. These have achieved large sample sizes and several have measured multiple QTs, but the twin design is substantially less powerful than sibship designs in which larger family units are recruited. Finally, there are a few pedigree-based studies which have been conducted in special genetic isolate populations. These have typically measured QTs but have been of relatively small size due to the population constraints in these unique populations. Thus GS:SFHS complements the recent large population-based study and biobank initiatives and the existing family based collections. Despite its focus on large sample size it may still be helpful to consider combining pedigree data with other international datasets to acquire additional power for linkage based approaches.

In summary, the family-based design of this study will permit both linkage and family-based association study approaches and the large sample size will give power to detect moderate genetic effects on QT levels [24,26]. The QTs studied underlie diseases which account for most of the burden of disease in Scotland.

### Current and future work

GS:SFHS is actively recruiting to the Phase 1 in both Tayside and Glasgow. It is intended that data from these participants will contribute to the main study database. A review of the Phase 1 will provide information on, among other factors, recruitment and participation rates, pedigree size and nature (and therefore power calculations), and planning for Phase 2 of the study across the whole of Scotland. These data will be written up for publication as the study progresses [9].

## Competing interests

SHR holds patents on genetic markers for osteoporosis susceptibility. There are no competing interests for any other author.

## Authors' contributions

All authors contributed to the writing of the study protocol, in an iterative manner. The main text was written by BHS and HC, with comments and amendments made by all authors, who have each read and approved the current version of the paper.

## Pre-publication history

The pre-publication history for this paper can be accessed here:


